# Digital Health Interventions for Adults With Type 2 Diabetes: Qualitative Study of Patient Perspectives on Diabetes Self-Management Education and Support

**DOI:** 10.2196/jmir.8439

**Published:** 2018-02-20

**Authors:** Kingshuk Pal, Charlotte Dack, Jamie Ross, Susan Michie, Carl May, Fiona Stevenson, Andrew Farmer, Lucy Yardley, Maria Barnard, Elizabeth Murray

**Affiliations:** ^1^ Department of Primary Care and Population Health University College London London United Kingdom; ^2^ Department of Psychology University of Bath Bath United Kingdom; ^3^ Centre for Behaviour Change University College London London United Kingdom; ^4^ Faculty of Health Sciences University of Southampton Southampton United Kingdom; ^5^ Nuffield Department of Primary Care Health Sciences University of Oxford Oxford United Kingdom; ^6^ Department of Psychology University of Southampton Southampton United Kingdom; ^7^ Department of Diabetic Medicine Whittington Hospital London United Kingdom

**Keywords:** diabetes mellitus, type 2, self-management, patient education, eHealth, mHealth, qualitative research

## Abstract

**Background:**

The prevalence of type 2 diabetes is increasing globally, and health services in many countries are struggling with the morbidity, mortality, and costs associated with the complications of this long-term condition. Diabetes self-management education (DSME) and behavioral support can reduce the risks of developing diabetes-related complications and improve glycemic control. However, their uptake is low. Digital health interventions (DHI) can provide sustained support and may overcome challenges associated with attending diabetes self-management sessions. They have the potential for delivery at multiple locations at convenient times, anonymity, and presentation of content in attractive and tailored formats. This study investigates the needs and wants of patients with type 2 diabetes to inform the development of digital self-management education and support.

**Objective:**

The objective of this study was to explore patient perspectives on unmet needs for self-management and support and the role of DHI in adults living with type 2 diabetes.

**Methods:**

This study used a qualitative approach based on data generated from 4 focus groups with 20 patients.

**Results:**

The data generated by the focus groups illustrated the significant burden that the diagnosis of diabetes places on many patients and the negative impacts on their emotional well-being, work, social life, and physical health. Although patients’ experiences of the health care services varied, there was agreement that even the best services were unable to meet all users’ needs to support the emotional regulation, psychological adjustment, and behavioral changes needed for successful self-management.

**Conclusions:**

By focusing on medical management and information provision, existing health care services and education programs may not be adequately meeting all the needs of patients with type 2 diabetes. DHIs have the potential to improve access to DSME and behavioral support and extend the range of content offered by health services to fit with a wider range of patient needs. Features that could help DHIs address some of the unmet needs described by participants in this study included placing an emphasis on emotional and role management, being available at all times, having up-to-date evidence-based guidance for patients, and providing access to peer-generated and professional advice.

## Introduction

### The Potential for Digital Health Interventions for Self-Management Education in Type 2 Diabetes

The prevalence of type 2 diabetes is increasing globally, and health services in many countries are struggling with the morbidity, mortality, and costs associated with the complications of this long-term condition [1]. Diabetes self-management education (DSME) is important as it can reduce the risks of developing diabetes-related complications and improve glycemic control, at least in the short term [2-4]. However, uptake of DSME and behavioral support is low: in England, less than 10% of newly diagnosed people with type 2 diabetes have been recorded as attending structured education [5], and in the United States, less than half of the patients have been found to receive DSME [6]. Barriers to attending DSME include inconvenience, fear of stigma, and a lack of knowledge about the potential benefits [7]. Education and behavioral support requirements by patients extend beyond initial DSME, and there is often a need for ongoing diabetes self-management support beyond educational courses in the light of patients’ evolving needs [8]. Digital health interventions (DHI) can provide sustained support and may overcome challenges associated with attending DSME. They have the potential for delivery at multiple locations at convenient times, anonymity, and presentation of content in attractive and tailored formats [9].

Examples of features used in existing interventions to improve the user experience include making fonts consistent, using logos and pictures, using bold to emphasize key points and improve design, repeatedly emphasizing the basic structure of the site and the program, including a screencast video to demonstrate the site, removing unhelpful jargon and terminology, managing expectations about the site in general, and personalizing the “source” by providing details about the development team [10]. User-expressed needs about the detail and degree of tailoring of information provision varies considerably, and it may not be possible to have a consensus on a format that satisfies all users [11].

### Potential Problems With Digital Health Interventions 

Challenges with DHIs include low levels of uptake in the target population with high levels of attrition and reduced user engagement over time [12]. The literature suggests there are two main approaches to improving uptake and maintaining ongoing engagement: (1) maximizing acceptability and usability of the intervention itself so patients *want* to use it [13,14] and (2) providing a degree of human support or facilitation so that patients are able to use it [15]. User engagement with interventions can be influenced by user perceptions [14] and intervention design [13]. User perceptions have both cognitive and affective elements [16]. Proposed cognitive drivers for user engagement include efficiency (ease of finding what users are looking for), effectiveness (impact of use), and trustworthiness. Important affective elements have been identified as enjoyment and interest [17]. Enjoyment can be defined as a general positive disposition and liking of media content [18], whereas interest motivates learning about something new and complex [19]. Users need to be interested to initially visit the website and then need to enjoy using the intervention to stay engaged [20]. With regard to understanding attrition, revisiting a website is associated with higher levels of education, being older, and a positive affective user experience [21]. Effective interventions therefore need to be interesting, enjoyable, and useful for patients. Qualitative research that explores the patient perspective can be a key tool to inform the development of such interventions.

### Study Design

This study describes qualitative research that was conducted with patients to identify user requirements before the development of a new Web-based self-management intervention for adults with type 2 diabetes called HeLP-Diabetes [22]. HeLP-Diabetes was developed as part of a 5-year National Institute for Health Research (NIHR) Programme Grant for Applied Research and provides comprehensive self-management support that includes health information, behavior change support, emotional support, self-monitoring tools, and access to online peer support. It can be used by adults with type 2 diabetes who are able to understand written English at any stage of their diabetes journey, and has been shown to be effective in improving diabetes control [23].

User requirements were conceptualized as “needs” and “wants,” where “needs” were features that were expected to achieve therapeutic benefit [24,25], whereas “wants” were features that users (patients) desired in an intervention and that were likely to make them want to return to the program.

The theoretical basis for understanding the *needs* of people living with type 2 diabetes was based on Corbin and Strauss’s model of the work of living with chronic illness [26]. Their study has informed much of the subsequent literature on self-management [27]. Corbin and Strauss described three types of work when living with a chronic illness: illness work, everyday life work, and biographical work. These tasks have also been described as medical management, emotional management, and role management [28]. Illness-related work consists of the tasks of managing treatment regimens, preventing and managing crises, symptom management, and diagnosis-related work. Everyday work describes the mundane work of everyday living and includes the sentimental work of managing emotions and relationships. Biographical work describes the work done in extracting meaning from life experiences and creating personal socially constituted identities as patients, parents, spouses, partners, professionals, or friends. Being diagnosed with a chronic illness can have a significantly disruptive impact on a person’s biographical narrative [29,30].

As mentioned previously, support or facilitation in using DHI can help increase use and potential impact of such interventions. Most of the data on the effects of providing human support or facilitation for DHI come from mental health, where studies on Internet cognitive behavioral therapy demonstrate that including therapist support significantly improves engagement with Internet cognitive behavioral therapy programs and their effectiveness [15,31]. In these studies, therapist input has been limited to encouraging engagement with the program, rather than providing therapy. However, human support, and in particular, health professional support, is an expensive and scarce resource, so it is important to determine how much human input is required and who should provide it.

This study was undertaken to determine patient needs and wants for a DHI to support self-management in patients living with type 2 diabetes.

## Methods

### Ethical Approval

The study was reviewed by the North West London Research and Ethics Committee (REC reference 10/H0722/86).

### Recruitment

English-speaking adults in England with type 2 diabetes were recruited for this study. Printed leaflets and posters were distributed to general practitioner (GP) surgeries and local diabetes support groups across London. An advertisement was placed in the *Diabetes Balance* magazine of Diabetes UK. Online recruitment included an advert on the Diabetes UK website, a local council website, ethnic minority forums, and other diabetes forums. Respondents were sent an information sheet and consent form and were invited to complete a questionnaire that was used to recruit a maximum variation sample. Factors that the literature suggested were likely to influence wants and needs included demographic factors (eg, age, gender, ethnicity, and first language), clinical factors (eg, duration of diabetes, current treatment, presence or absence of diabetes-related complications, and previous experience of self-management programs), and factors related to health and computer literacy (such as educational attainment, previous experience with computers, and access to the Internet) [32-35]. Participants were purposively sampled to vary across these characteristics.

### Data Collection

Four focus groups with 3 to 6 participants were held in a community center in London. Focus groups were chosen as they encourage interactions between participants and allow ideas to be generated, reflected on, and debated by participants [36,37]. They were facilitated by two or three researchers from the team (CD, KP, EM, and FS), audio-recorded, and transcribed verbatim. Previous reviews of patient information materials and websites provided the basis for the structure of the focus groups [38,39]. At the start of the focus group, participants were shown 3 examples of existing websites that support diabetes self-management and asked to explore them. These had been selected by the research team to demonstrate the range of interventions and component parts available. They varied in terms of content, complexity, tone, navigation, and presence of interactive features such as forums, Ask the Expert, or self-monitoring tools. The websites were selected based on a number of criteria: up-to-date, accredited by national diabetes organizations (eg, American Diabetes Association or Diabetes UK), and written in English. The topic guide was piloted in an interview by one researcher (CD) to ensure it stimulated discussion across the target areas.

The 4 focus groups were held in 2 community centers. The facilities had computer access and rooms suitable for small group discussions. A venue outside health care settings was chosen to put participants at ease and to minimize the impact of the power differentials that might be created in such settings from participants taking on the role of a patient.

Each focus group was run by 2 to 3 researchers and lasted up to 4 hours in total. The first 15 min was allocated to welcoming participants and introductions, followed by up to 90 min exploring the 3 websites. This was followed by a 30-min break and then 90 min of group discussion. Having more than one researcher was helpful as they took a more observational role to monitor the interactions between facilitator and participant and could pick up on undue prompting or dominance from the main facilitator [40]. Having nonmedical cofacilitators helped monitor for dynamics that would limit the data generated from the focus groups if the facilitator took on the role of the “expert” and inhibited participant discussions [40]. Discussions were semistructured with a list of topics to be covered (not necessarily in a defined order) during the session.

Data collection continued until no new data emerged regarding content and design or patient-defined wants and needs for self-management.

### Data Analysis

Analysis was conducted by a multidisciplinary team. Transcripts were independently read by five authors (CD, KP, JR, EM, and FS), and emerging themes were discussed at a multidisciplinary meeting. As described previously, the underlying sociological theory guiding the analysis of patient needs was Corbin and Strauss’s study on living with a chronic illness. The main constructs of the model were used to sensitize and encourage a holistic perspective that explored the impact of type 2 diabetes mellitus on people’s day-to-day activities, relationships, and emotions (everyday life work); the burden of having to take medicines or make lifestyle changes for the medical management of the condition (illness work); and the disruption or changes to the roles that patients played within their families and at work (biographical work). This model was not used to define a priori codes or categories, but was used as a sensitizing tool to organize codes generated using inductive thematic analysis. Two authors (EM and KP) coded the transcripts and mapped themes onto the Corbin and Strauss’s model. The mapping was then discussed and agreed upon in a meeting between the 5 authors (CD, JR, FS, KP, and EM). The resulting structure was used to determine patient needs and wants for a DHI. None of the themes developed inductively fell out with the Corbin and Strauss’s model.

Illustrative extracts of the data are presented in the Results, with identification by focus group number and participant number together with age, gender, ethnicity, duration of diabetes, and computer experience.

Atlas Ti (version 6.2, Scientific Software Development, Berlin, Germany) was used to manage the transcripts and coding and to facilitate the final data analysis.

## Results

### Participant Characteristics

The demographics of the 20 participants who took part in the pilot interview and 4 focus groups are summarized in Table 1.

Just over half the participants were male with a mean age of nearly 57 years. Almost half were retired and over half had degree-level qualifications. Moreover, 70% (14/20) of participants were white; the remaining participants described their ethnicity as black, Asian, or other. Time since diagnosis ranged from 3 months to 36 years. An overwhelming majority of participants had home Internet access, and most had used the Internet to look up information about diabetes. In addition, 60% (12/20) of participants had been on a diabetes self-management course, but most had never used a computer-based self-management program.

The data mapped easily onto the Corbin and Strauss’s model, with the resultant themes and subthemes summarized in Table 2.

### Participant-Defined Health Needs

#### Challenges With Role Management

Many participants described a significant and constant burden that they experienced as a result of the diagnosis. Some participants felt a sense of loss at diagnosis, and the identity constructed around being a patient with type 2 diabetes was quite negative and associated with poor health, stigma, and shame:

Facilitator: you’ve described it [diagnosis of diabetes] being like bereavement, and a lot of people say that, because you are, in a sense; you're grieving for the loss of your...?

PT5: Liberty. Freedom.

Facilitator: Is that what it is? Right.

PT10: Health.

PT5: Yes.

PT5:Well, it’s more to do with the mortality business, isn't it? Without wanting to sound grim, but...

PT5: male, 55 years old, white British, 5 years since diagnosis; PT10: male, 70 years old, white British, 6 months since diagnosis; focus group 1

Participants reported a perception that other people blamed them for their illness, and that the relationship between lifestyle and type 2 diabetes led to stigmatization of people who developed diabetes:

Usually people say, oh, you must have had a bad lifestyle, something or whatever, which may be true sometimes, but it’s not the only reason, so...

PT19: female, 64 years old, white British, 36 years since diagnosis; focus group 3

Many of the participants described how the demands of an illness that required them to take medication and eat regularly made it impossible for them to carry on with the work they had previously been doing:

You know, and again, they took me off shifts, because I couldn’t remember if I’d taken my pills one week, you know? One week, I’m working early and next week, I’m working late, and then I’m working nights, and I used to go, I can’t remember if I’ve taken them or not. My manager said, that’s no use, is it?

PT17: male, 54 years old, white British, 8 years since diagnosis; focus group 3

#### Managing the Emotional Burden of Diabetes

Participants reported experiencing strong negative emotions, which they found difficult to manage. Participants frequently reported experiencing depression, anger, frustration, and guilt.

Low, angry, frustrated. Everything. Because, you know, sometimes you're frustrated because the doctor hasn't told you what you want to hear. Or you're angry with the world, and you take it out on your children, your partners, everybody. And then you've got the depression that takes you down, because you're just thinking one thing after another.

PT11: Female, 51 years old, black (Caribbean), 10 years since diagnosis; focus group 2

The impact of dietary changes on participants’ social and family lives was also experienced as a difficultly. Some participants found it too hard to keep to their planned diet when out with friends or family, and would simply try to manage the consequences, whereas others would try to adhere to the changes they had instituted, but reported negative reactions from their families:

Because we're talking about food; I mean, I go to my family, and when I say I can't eat that food, they usually think that's disrespecting them, so you've got all that as well to deal with.

PT11: Female, 51 years old, black (Caribbean), 10 years since diagnosis; focus group 2

**Table 1:** Demographics of participants.

Characteristic		Value
**Gender, n (%)**		
	Male	12 (60)
	Female	8 (40)
Age in years, mean (range)		56.8 (36-77)
**Employment status, n (%)**		
	Employed	5 (25)
	Not working but looking for work	2 (10)
	Retired	8 (40)
	Retired (semi)	1 (5)
	Not working and not looking for work	2 (10)
	Other—full time student	1 (5)
	Other—volunteer	1 (5)
**Education, n (%)**		
	School leaver	4 (20)
	A Level	5 (25)
	Degree	11 (55)
**Ethnicity**		
	White British	14 (70)
	Black (African, Caribbean, and other)	4 (20)
	Asian (Indian)	1 (5)
	Other (Iranian)	1 (5)
**Duration of diabetes, n (%)**		
	<1 year	2 (10)
	1-5 years	7 (35)
	6-10 years	5 (25)
	>10 years	6 (30)
**Diabetes management, n (%)**		
	Diet only	3 (15)
	Diet + tablets	10 (50)
	Diet + tablets + liraglutide injection	1 (5)
	On insulin	6 (30)
**Home Internet access, n (%)**		
	Yes	19 (95)
	No	1 (5)
**Attended diabetes education, n (%)**		
	Yes	12 (60)
	No	8 (40)
**Used the Internet to look up diabetes-related information, n (%)**		
	Yes	17 (85)
	No	3 (15)
**Used a computer self-management intervention before, n (%)**		
	No	16 (80)
	Yes	2 (10)
	Yes (own spreadsheets)	2 (10)

#### Medical Management: Problems With Existing Health Services

Although some participants were very appreciative of the care they had received from the health service, this was not a universal experience, with many participants reporting difficulties with access to health care professionals, lack of interest or expertise in staff, and an increasing sense of a “tick box” culture, where problems were recorded but not addressed. Even participants who were positive about their care reported unease about taking up time in consultations:

I've been very fortunate with my practice in [location] because they've given me a huge amount of support actually in terms of information gathering. But I understand that you've only got to be a couple of miles down the road and you get nothing at all. And even if you ask the questions, the doctors feel that you're taking up their time, and in fact that's true of all doctors, I appreciate that.

PT10: male, 70 years old, white British, 6 months since diagnosis; focus group 1

I'm asked that, once a year, that question, do you feel depressed? Yes. Next question. It’s not like, what are you going to do about it? And when I see the nurse, every six months, she just says, are you exercising? And she ticks a little box if I say yes or no. And that’s it.

PT6: male, 55 years old, black (African), 10 years since diagnosis; focus group 1

Participants had experience of poor quality information that was difficult to understand and not relevant to their personal needs. They were clear that they wanted to have access to detailed information in case it was needed, but controlling the flow of information was very important to avoid “information overload.” Patients wanted access to in-depth information when it was relevant to them. An example of this included information about abnormal test results or dietary advice that took into account personal circumstances. Some participants struggled with complex information, for example, understanding nutritional content and guidance about recommended daily allowances:

...but sometimes it’s a question of having too much information and you can’t take it all on board and you can’t make all the changes overnight. [PT20: female, 41 years old, white British, 5 years since diagnosis; pilot interview] And where are the tools that help me to understand it? You know, I'm looking at carbs and sugar, and it's all very confusing and highly complicated.

PT16: male, 58 years old, white British, 4 months since diagnosis; focus group 4

Participants emphasized the need for self-management to be integrated with health care professional management and thought it would be beneficial to have access to their electronic medical records (EMR). They thought it would be important to correct misinformation and have the correct information to share with their multiple different health care providers, such as opticians, podiatrists, dentists, and emergency doctors. However, participants were keen to have control over their information, and to decide what to share with whom:

I think it might be useful to correct things, if you find anything that's been recorded incorrectly, that at the moment, you've got no idea if there's anything…anything's wrong or not.

PT16: male, 58 years old, white British, 4 months since diagnosis; focus group 4

I'm a great believer in being able to access your own records, and also having...the worst thing is, when you go along to A&E, and they say to you...they might turn around to you and say, oh when were you diagnosed? You know, and you have to start from...the whole story from the beginning.

PT7: female, 65 years old, white British, 3 years since diagnosis; focus group 4

**Table 2:** Mapping of patient needs onto Corbin and Strauss’s model of living with a chronic illness. GP: general practitioner. DSME: diabetes self-management education. EMR: electronic medical record.

Level 1 and Level 2		Level 3
**Life work and emotional management**		
	Negative emotions associated with living with type 2 diabetes	Burn-out
		Denial
		Indifference
		Depression
		Anger
		Frustration
		Self-blame
		Guilt
		Shame
	Triggers for negative emotions	Food
		Seeking medical help
		Lack of understanding from family members
		Intrusive comments from family members
	Strategies for keeping a positive outlook	Maintaining optimism
		Accept limits on control
		Treat depression
	Sources of support	Caring family members
		Peers
**Illness work and medical management**		
	Barriers posed by the health care system	“Tick-box” consultations
		Conflicting advice
		Professionals not keeping up to date
		Difficulty getting appointments with GP
		Difficult accessing DSME
		Poor quality information (too much, too little, too complicated, not relevant)
	Enablement by the health care system	Supportive doctors and nurses
		Taking time to explain results
		Timely access to DSME
	Low priority of illness work	Lack of time
	Features patients want from access to EMR	Access to blood results
		Access to a medical summary
		Transparency and being able to correct errors
		Control data sharing
**Biographical work and role management**		
	Negative self-image	Feeing bereaved of loss of health
		Deserving punishment
		Feeling like a criminal
		Stigma of diagnosis
	Changes in parent role	Dependence on children
	Changes in working roles	Lack of support for making adaptations to work roles
		Impact of changing needs of patient role on working role

#### What Participants Wanted From a Digital Health Intervention: Content and Design Features

Participants were clear that digital interventions should address all aspects of living with diabetes, including diet, physical activity, taking medicines, working with health care professionals, managing difficult emotions, and handling interactions at work, social occasions, and with friends and family. Of those, food and nutrition therapy were of most interest. Participants were also interested in hearing about alternative medicine and the opinions of peers.

They wanted information about diabetes, including how it is caused and how it affects the body; available treatments, including goals of treatment, pros and cons of each treatment, and potential side effects; and access to a suite of resources. They wanted the program to be a “one-stop shop,” which they could turn to at times of need. There were clear tensions between wanting and needing information on the one hand and, on the other, not wanting to be overwhelmed with “bad news.” They stated it was vital that the information was presented in a positive fashion, with an emphasis on what can be done to prevent complications, acknowledging that diabetes can be hard to control and avoiding “victim blaming” when things do go wrong:

I suppose I’d want something that was a bit, kind of, an A to Z of one’s life.

PT20: female, 41 years old, white British, 5 years since diagnosis; pilot interview

So that's the big problem, it seems to me. The mainstream medical opinion seems to be all doom and gloom...If you just put that diabetes is such and such but can be controlled or managed or whatever word you want to use, through very simple means, I think that's a huge relief to people.

PT10: male, 70 years old, white British, 6 months since diagnosis; focus group 1

I think you've always got to look at the positive side of your illness. But yes, you're always going to have a negative side, and sometimes you've got to have a funny side...

PT8: female, 46 years old, white British, 16 years since diagnosis; focus group 1

Textboxes 1 and 2 summarize participants’ views on the content and design features they wanted to see in a DHI supporting self-management in type 2 diabetes.

#### Health Professional Facilitation and Other Elements That Might Engage Users

Participants wanted self-management support programs to be integrated into their general medical care, rather than stand-alone. They wanted to work with their health professionals to obtain good health outcomes. Participants were therefore strongly in favor of their health care professionals helping users register on a DHI, showing people how to use it, and discussing their use of the program in diabetes-related consultations, but they were skeptical as to whether this would be possible:

I think it would be a good thing but I can’t see many people doing it. I know obviously they, to even ring your GP surgery normally to make an appointment can be very tiresome for a lot of people. You can’t get through.

PT8: female, 46 years old, white British, 16 years since diagnosis; focus group 1

Participants suggested the following potential features that might encourage users to access a DHI on an ongoing basis: regularly adding new content; articles about latest research findings; regular emails; the use of video, forums, and interactive tools; and the ability to use administrative functions such as booking appointments with health care professionals.

**Summary of the range of content desired by participants in this study.**

Medical information

- Medication
- Side effects
- Hypoglycemia
- News and research

Dietary advice

- Controlling blood glucose
- Weight loss
- Controlling cholesterol
- Understanding food
- Recipe ideas

Physical activity

- Benefits of exercise
- Advice about weight loss
- Self-monitoring tools
- Easy-to-do physical activities
- Resources for group activities

Alternative medicine

- Relaxation therapy and stress reduction
- Complementary therapies

Peer support

- Advice from peers
- Emotional support
- Social comparisons
- Role models
- Not much enthusiasm for social media

Pregnancy

Safe conception and what to do if pregnant

Practical advice

- Travel
- Insurance
- Financial advice, for example, benefits

Information about health services

- Services that patients should have access to
- Local support groups
- Telephone support lines

**Design features desired by participants in this study.**

Design and navigation

- Easy to use
- Clear, concise, and consistent
- Minimize scrolling
- Use videos
- Pages can be printed in black and white
- Have interactive features like quizzes
- Provide self-monitoring tools
- Act as a central hub for all diabetes-related queries with links to other resources

Language and tone

- Accessible
- Use medical terminology where needed, but provide definitions and explanations
- Be encouraging and supportive
- Not shy away from difficult truths
- Have a sense of humor

Trust

- Thorough proofreading
- No advertising
- Working links to and from good websites
- Use trusted brands

Avoid irritants

- Poor design
- Not relevant or localized
- Out of date
- Boring or static

Keeping users engaged

- Regularly adding new content
- Articles about latest research findings
- Regular emails
- Use of video and interactive tools
- Booking appointments

## Discussion

### Principal Findings

The participants in this study described a range of unmet needs for supporting their self-management efforts and identified a number of potential ways that DHI could help them. The features of DHIs desired by participants included specific content relating to diabetes (eg, hypoglycemia, medication side-effects, weight loss, and physical activity) and emphasized the strong desire of participants for reliable and accessible dietary advice. Patients in the United Kingdom have good access to primary care doctor and nursing support with structured templates to help standardize diabetes care. However, where patient self-management needs extend beyond the remit of these structures that often focus on using medication to optimize glycemic control, it can be difficult to get support.

A DHI could help overcome some of the barriers to self-management currently posed by limitations of existing health care systems. An evidence-based, well-written, up-to-date DHI could be available 24/7, and could improve access to high-quality information, DSME, and behavioral support where patients are not able to access face-to-face services that provide tailored information and help with behavior changes such as increasing physical activity, dietary change, and weight loss.

Access for patients to their electronic medical record via the DHI was seen as a potential benefit that could engage patients, especially if access to these systems allowed administrative functions such as booking medical appointments. Technology-based prompts have been shown to have positive effects on engagement with other DHIs [10], and participants in this study reported that regular emails would be an acceptable strategy for increasing intervention use.

The DHI developed as a result of these data, HeLP-Diabetes, was effective in improving diabetes control for users. We believe a key reason for this was the careful attention paid during the development phase to users’ wants and needs, and we commend this approach to others. The data generated during the focus groups were underpinned by a strong sense of the burden that the diagnosis of diabetes placed on participants, which had negative impacts on their emotional well-being, work, social life, and physical health. This finding fitted with our overall theoretical framework, based on the Corbin and Strauss’s model, and underlined the importance of ensuring that the self-management program addressed the three key tasks of medical, emotional, and role management. Although participants’ experiences of the health care services varied, participants reported difficulties getting the information and patient-centered care they needed to support self-management when consultations with health professionals were too time-pressured or protocol-driven to accommodate individual patient needs.

The strengths of this study included the use of focus groups and semistructured topic guides, which allowed participants to raise their own concerns and determine the direction and content of the discussions. The use of a sociological model [17] encouraged a holistic approach to the data that provided a broader perspective on self-management than usual bio-medical definitions of DSME.

### Limitations

The main limitation was that, although we were successful in recruiting a sample that was diverse in terms of ethnicity, duration and treatment of diabetes, and gender, the participants who volunteered for the study were relatively well-educated and computer-literate and the sample may have over-represented patients who were motivated and actively engaged in self-management. People with lower health literacy and from other cultures may have different needs that were not explored in this study. The majority of participants had also not had any previous experience of using a computer-based self-management program. The risks of using a naïve population are that interventions might not be optimized for the needs of potential users who are most comfortable using Web-based tools and suggestions for intervention development would not be grounded in a detailed understanding of existing interventions. However, the advantage of using a “naïve” population is potentially better generalizability with the needs and preferences representing the general population rather than early adopters who might be more technically savvy. The exponential growth of the Internet and mobile phone use illustrates how useful and user-friendly technology can have a mass-market impact; thus, exploring the needs of a study population that represents a more generalizable population beyond early adopters would be important in maximizing the potential benefits of such an intervention. Well-designed DHIs for diabetes have been shown to engage and benefit users with a wide range of health literacy, and findings can be generalizable to a wide range of users [41].

### Comparison With Prior Work

There were many areas where a DHI could help address the unmet needs described above. There have been a number of systematic reviews of DHIs in type 2 diabetes, including narrative syntheses, meta-analyses, and meta-ethnographies [9,42-44]. The interventions described in these reviews have tended to focus on improving the medical management of type 2 diabetes through information provision and behavior change support [45-49]. However, for participants in this study, the “work” of emotional and role management was more important for them than most aspects of medical management other than food and diet. Previous studies have highlighted inconvenience and poor motivation as reasons for poor engagement with DSME and DHI for diabetes [7,49]. This study suggests that another important aspect might be a potential mismatch between the content and emphasis of existing DSME interventions and patient-defined needs and priorities for diabetes self-management. Increasing the focus on emotional management for such interventions also has clinical importance as cross-sectional and prospective evidence suggests that changes in diabetes-related distress correlate with changes in glycemic control, possibly through changes in adherence with medication, whereas depressive symptoms are correlated with self-management behavior [50,51].

### Conclusions

By focusing on medical management and information provision, existing health care services and education programs may not be adequately meeting the needs of patients with type 2 diabetes. DHIs have the potential to help improve access to DSME and extend the range of content offered by health services to meet a wider range of patient needs. The features of a DHI that could address the unmet needs described by participants in this study included an emphasis on emotional and role management, being available 24/7, having up-to-date evidence-based guidance for patients, and providing access to peer-generated and professional advice. The findings of this study have been used in the development of an effective DHI for adults with type 2 diabetes, called HeLP-Diabetes, which has been evaluated in a randomized controlled trial and implementation study and has been shown to be acceptable to a wide range of users [23].

## Abbreviations

DHI: digital health intervention
DSME: diabetes self-management education
GP: general practitioner
NIHR: National Institute for Health Research
